# Factors in the emergence of infectious diseases.

**DOI:** 10.3201/eid0101.950102

**Published:** 1995

**Authors:** S S Morse

**Affiliations:** Rockefeller University, New York, NY 10021-6399, USA. morse@rockvax.rockefeller.edu

## Abstract

"Emerging" infectious diseases can be defined as infections that have newly appeared in a population or have existed but are rapidly increasing in incidence or geographic range. Among recent examples are HIV/AIDS, hantavirus pulmonary syndrome, Lyme disease, and hemolytic uremic syndrome (a foodborne infection caused by certain strains of Escherichia coli). Specific factors precipitating disease emergence can be identified in virtually all cases. These include ecological, environmental, or demographic factors that place people at increased contact with a previously unfamiliar microbe or its natural host or promote dissemination. These factors are increasing in prevalence; this increase, together with the ongoing evolution of viral and microbial variants and selection for drug resistance, suggests that infections will continue to emerge and probably increase and emphasizes the urgent need for effective surveillance and control. Dr. David Satcher's article and this overview inaugurate Perspectives, a regular section in this journal intended to present and develop unifying concepts and strategies for considering emerging infections and their underlying factors. The editors welcome, as contributions to the Perspectives section, overviews, syntheses, and case studies that shed light on how and why infections emerge, and how they may be anticipated and prevented.


### Factors in the Emergence of Infectious Diseases

**Stephen S. Morse, Ph.D.**
The Rockefeller University, New York, NewYork, USA

*"Emerging" infectious diseases can be defined as infections that have newly appeared in a population or have existed but are rapidly increasing in incidence or geographic range. Among recent examples are HIV/AIDS, hantavirus pulmonary syndrome, Lyme disease, and hemolytic uremic syndrome (a foodborne infection caused by certain strains of* Escherichia coli*). Specific factors precipitating disease emergence can be identified in virtually all cases. These include ecological, environmental, or demographic factors that place people at increased contact with a previously unfamiliar microbe or its natural host or promote dissemination. These factors are increasing in prevalence; this increase, together with the ongoing evolution of viral and microbial variants and selection for drug resistance, suggests that infections will continue to emerge and probably increase and emphasizes the urgent need for effective surveillance and control. Dr. David Satcher's article and this overview inaugurate "Perspectives," a regular section in this journal intended to present and develop unifying concepts and strategies for considering emerging infections and their underlying factors. The editors welcome, as contributions to the Perspectives section, overviews, syntheses, and case studies that shed light on how and why infections emerge, and how they may be anticipated and prevented.*

Infectious diseases emerging throughout history have included some of the most feared plagues of the past. New infections continue to emerge today, while many of the old plagues are with us still. These are global problems (William Foege, former CDC director now at the Carter Center, terms them "global infectious disease threats"). As demonstrated by influenza epidemics, under suitable circumstances, a new infection first appearing anywhere in the world could traverse entire continents within days or weeks.

We can define as "emerging" infections that have newly appeared in the population, or have existed but are rapidly increasing in incidence or geographic range (1,2). Recent examples of emerging diseases in various parts of the world include HIV/AIDS; classic cholera in South America and Africa; cholera due to *Vibrio cholerae* O139; Rift Valley fever; hantavirus pulmonary syndrome; Lyme disease; and hemolytic uremic syndrome, a foodborne infection caused by certain strains of *Escherichia coli* (in the United States, serotype O157:H7).

Although these occurrences may appear inexplicable, rarely if ever do emerging infections appear without reason. Specific factors responsible for disease emergence can be identified in virtually all cases studied (2-4). Table 1 summarizes the known causes for a number of infections that have emerged recently. I have suggested that infectious disease emergence can be viewed operationally as a two-step process: 1) Introduction of the agent into a new host population (whether the pathogen originated in the environment, possibly in another species, or as a variant of an existing human infection), followed by 2) establishment and further dissemination within the new host population ("adoption") (4). Whatever its origin, the infection "emerges" when it reaches a new population. Factors that promote one or both of these steps will, therefore, tend to precipitate disease emergence. Most emerging infections, and even antibiotic-resistant strains of common bacterial pathogens, usually originate in one geographic location and then disseminate to new places (5).

Regarding the introduction step, the numerous examples of infections originating as zoonoses (7,8) suggest that the "zoonotic pool"—introductions of infections from other species—is an important and potentially rich source of emerging diseases; periodic discoveries of "new" zoonoses suggest that the zoonotic pool appears by no means exhausted. Once introduced, an infection might then be disseminated through other factors, although rapid course and high mortality combined with low transmissibility are often limiting. However, even if a zoonotic agent is not able to spread readily from person to person and establish itself, other factors (e.g., nosocomial infection) might transmit the infection. Additionally, if the reservoir host or vector becomes more widely disseminated, the microbe can appear in new places.

Address for correspondence: Stephen S. Morse, The Rockefeller University, 1230 York Avenue, Box 120, New York, NY 10021-6399, USA; fax 212-327-7172; e-mail morse@rockvax.rockefeller.edu.

**Table 1. Recent examples of emerging infections and probable factors in their emergence**

| Infection or Agent | Factor(s) contributing to emergence |
|---|---|
| **Viral** | |
| Argentine, Bolivian hemorrhagic fever | Changes in agriculture favoring rodent host |
| Bovine spongiform encephalopathy (cattle) | Changes in rendering processes |
| Dengue, dengue hemorrhagic fever | Transportation, travel, and migration; urbanization |
| Ebola, Marburg | Unknown (in Europe and the United States, importation of monkeys) |
| Hantaviruses | Ecological or environmental changes increasing contact with rodent hosts |
| Hepatitis B, C | Transfusions, organ transplants, contaminated hypodermic apparatus, sexual transmission, vertical spread from infected mother to child |
| HIV | Migration to cities and travel; after introduction, sexual transmission, vertical spread from infected mother to child, contaminated hypodermic apparatus (including during intravenous drug use), transfusions, organ transplants |
| HTLV | Contaminated hypodermic apparatus, other |
| Influenza (pandemic) | Possibly pig-duck agriculture, facilitating reassortment of avian and mammalian influenza viruses* |
| Lassa fever | Urbanization favoring rodent host, increasing exposure (usually in homes) |
| Rift Valley fever | Dam building, agriculture, irrigation; possibly change in virulence or pathogenicity of virus |
| Yellow fever (in "new" areas) | Conditions favoring mosquito vector |
| **Bacterial** | |
| Brazilian purpuric fever (*Haemophilus influenzae*, biotype *aegyptius*) | Probably new strain |
| Cholera | In recent epidemic in South America, probably introduced from Asia by ship, with spread facilitated by reduced water chlorination; a new strain (type O139) from Asia recently disseminated by travel (similarly to past introductions of classic cholera) |
| *Helicobacter pylori* | Probably long widespread, now recognized (associated with gastric ulcers, possibly other gastrointestinal disease) |
| Hemolytic uremic syndrome (*Escherichia coli* O157:H7) | Mass food processing technology allowing contamination of meat |
| *Legionella* (Legionnaires' disease) | Cooling and plumbing systems (organism grows in biofilms that form on water storage tanks and in stagnant plumbing) |
| Lyme borreliosis (*Borrelia burgdorferi*) | Reforestation around homes and other conditions favoring tick vector and deer (a secondary reservoir host) |
| *Streptococcus*, group A (invasive; necrotizing) | Uncertain |
| Toxic shock syndrome (*Staphylococcus aureus*) | Ultra-absorbency tampons |
| **Parasitic** | |
| *Cryptosporidium*, other waterborne pathogens | Contaminated surface water, faulty water purification |
| Malaria (in "new" areas) | Travel or migration |
| Schistosomiasis | Dam building |

*Reappearances of influenza are due to two distinct mechanisms: Annual or biennial epidemics involving new variants due to antigenic drift (point mutations, primarily in the gene for the surface protein, hemagglutinin) and pandemic strains, arising from antigenic shift (genetic reassortment, generally between avian and mammalian influenza strains).

Bubonic plague transmitted by rodent fleas and ratborne hantavirus infections are examples.

Most emerging infections appear to be caused by pathogens already present in the environment, brought out of obscurity or given a selective advantage by changing conditions and afforded an opportunity to infect new host populations (on rare occasions, a new variant may also evolve and cause a new disease) (2,4). The process by which infectious agents may transfer from animals to humans or disseminate from isolated groups into new populations can be called "microbial traffic" (3,4). A number of activities increase microbial traffic and as a result promote emergence and epidemics. In some cases, including many of the most novel infections, the agents are zoonotic, crossing from their natural hosts into the human population; because of the many similarities, I include here vector-borne diseases. In other cases, pathogens already present in geographically isolated populations are given an opportunity to disseminate further. Surprisingly often, disease emergence is caused by human actions, however inadvertently; natural causes, such as changes in climate, can also at times be responsible (6). Although this discussion is confined largely to human disease, similar considerations apply to emerging pathogens in other species.

Table 2 summarizes the underlying factors responsible for emergence. Any categorization of the factors is, of course, somewhat arbitrary but should be representative of the underlying processes that cause emergence. I have essentially adopted the categories developed in the Institute of Medicine report on emerging infections (12), with additional definitions from the CDC emerging infections plan (13). Responsible factors include ecological changes, such as those due to agricultural or economic development or to anomalies in climate; human demographic changes and behavior; travel and commerce; technology and industry; microbial adaptation and change; and breakdown of public health measures. Each of these will be considered in turn.

Ecological interactions can be complex, with several factors often working together or in sequence. For example, population movement from rural areas to cities can spread a once-localized infection. The strain on infrastructure in the overcrowded and rapidly growing cities may disrupt or slow public health measures, perhaps allowing establishment of the newly introduced infection. Finally, the city may also provide a gateway for further dissemination of the infection. Most successful emerging infections, including HIV, cholera, and dengue, have followed this route.

Consider HIV as an example. Although the precise ancestry of HIV-1 is still uncertain, it appears to have had a zoonotic origin (9,10). Ecological factors that would have allowed human exposure to a natural host carrying the virus that was the precursor to HIV-1 were, therefore, instrumental in the introduction of the virus into humans. This probably occurred in a rural area. A plausible scenario is suggested by the identification of an HIV-2-infected man in a rural area of Liberia whose virus strain resembled viruses isolated from the sooty mangabey monkey (an animal widely hunted for food in rural areas and the putative source of HIV-2) more closely than it did strains circulating in the city (11). Such findings suggest that zoonotic introductions of this sort may occur on occasion in isolated populations but may well go unnoticed so long as the recipients remain isolated. But with increasing movement from rural areas to cities, such isolation is increasingly rare. After its likely first move from a rural area into a city, HIV-1 spread regionally along highways, then by long distance routes, including air travel, to more distant places. This last step was critical for HIV and facilitated today's global pandemic. Social changes that allowed the virus to reach a larger population and to be transmitted despite its relatively low natural transmissibility were instrumental in the success of the virus in its newfound human host. For HIV, the long duration of infectivity allowed this normally poorly transmissible virus many opportunities to be transmitted and to take advantage of such factors as human behavior (sexual transmission, intravenous drug use) and changing technology (early spread through blood transfusions and blood products) (Table 1).

### Ecological Changes and Agricultural Development

Ecological changes, including those due to agricultural or economic development, are among the most frequently identified factors in emergence. They are especially frequent as factors in outbreaks of previously unrecognized diseases with high case-fatality rates, which often turn out to be zoonotic introductions. Ecological factors usually precipitate emergence by placing people in contact with a natural reservoir or host for an infection hitherto unfamiliar but usually already present (often a zoonotic or arthropod-borne infection), either by increasing proximity or, often, also by changing conditions so as to favor an increased population of the microbe or its natural host (2,4). The emergence of Lyme disease in the United States and Europe was probably due largely to reforestation (14), which increased the population of deer and the deer tick, the vector of Lyme disease. The movement of people into these areas placed a larger population in close proximity to the vector.

Agricultural development, one of the most common ways in which people alter and interpose themselves into the environment, is often a factor

**Table 2. Factors in infectious disease emergence***

| Factor | Examples of specific factors | Examples of diseases |
|---|---|---|
| Ecological changes (including those due to economic development and land use) | Agriculture; dams, changes in water ecosystems; deforestation/reforestation; flood/drought; famine; climate changes | Schistosomiasis (dams); Rift Valley fever (dams, irrigation); Argentine hemorrhagic fever (agriculture); Hantaan (Korean hemorrhagic fever) (agriculture); hantavirus pulmonary syndrome, southwestern US, 1993 (weather anomalies) |
| Human demographics, behavior | Societal events: Population growth and migration (movement from rural areas to cities); war or civil conflict; urban decay; sexual behavior; intravenous drug use; use of high-density facilities | Introduction of HIV; spread of dengue; spread of HIV and other sexually transmitted diseases |
| International travel and commerce | Worldwide movement of goods and people; air travel | "Airport" malaria; dissemination of mosquito vectors; ratborne hantaviruses; introduction of cholera into South America; dissemination of O139 *V. cholerae* |
| Technology and industry | Globalization of food supplies; changes in food processing and packaging; organ or tissue transplantation; drugs causing immunosuppression; widespread use of antibiotics | Hemolytic uremic syndrome (*E. coli* contamination of hamburger meat), bovine spongiform encephalopathy; transfusion-associated hepatitis (hepatitis B, C), opportunistic infections in immunosuppressed patients, Creutzfeldt-Jakob disease from contaminated batches of human growth hormone (medical technology) |
| Microbial adaptation and change | Microbial evolution, response to selection in environment | Antibiotic-resistant bacteria, "antigenic drift" in influenza virus |
| Breakdown in public health measures | Curtailment or reduction in prevention programs; inadequate sanitation and vector control measures | Resurgence of tuberculosis in the United States; cholera in refugee camps in Africa; resurgence of diphtheria in the former Soviet Union |

*Categories of factors (column 1) adapted from ref. 12, examples of specific factors (column 2) adapted from ref. 13. Categories are not mutually exclusive; several factors may contribute to emergence of a disease (see Table 1 for additional information).

(Table 2). Hantaan virus, the cause of Korean hemorrhagic fever, causes over 100,000 cases a year in China and has been known in Asia for centuries. The virus is a natural infection of the field mouse *Apodemus agrarius*. The rodent flourishes in rice fields; people usually contract the disease during the rice harvest from contact with infected rodents. Junin virus, the cause of Argentine hemorrhagic fever, is an unrelated virus with a history remarkably similar to that of Hantaan virus. Conversion of grassland to maize cultivation favored a rodent that was the natural host for this virus, and human cases increased in proportion with expansion of maize agriculture (15). Other examples, in addition to those already known (2,15), are likely to appear as new areas are placed under cultivation.

Perhaps most surprisingly, pandemic influenza appears to have an agricultural origin, integrated pig-duck farming in China. Strains causing the frequent annual or biennial epidemics generally result from mutation ("antigenic drift"), but pandemic influenza viruses do not generally arise by this process. Instead, gene segments from two influenza strains reassort to produce a new virus that can infect humans (16). Evidence amassed by Webster, Scholtissek, and others, indicates that waterfowl, such as ducks, are major reservoirs of influenza and that pigs can serve as "mixing vessels" for new mammalian influenza strains (16). Pandemic influenza viruses have generally come from China. Scholtissek and Naylor suggested that integrated pig-duck agriculture, an extremely efficient food production system traditionally practiced in certain parts of China for several centuries, puts these two species in contact and provides a natural laboratory for making new influenza recombinants (17). Webster has suggested that, with high-intensity agriculture and movement of livestock across borders, suitable conditions may now also be found in Europe (16).

Water is also frequently associated with disease emergence. Infections transmitted by mosquitoes or other arthropods, which include some of the most serious and widespread diseases (18,19), are often stimulated by expansion of standing water, simply because many of the mosquito vectors breed in water. There are many cases of diseases transmitted by water-breeding vectors, most involving dams, water for irrigation, or stored drinking water in cities. (See "Changes in Human Demographics and Behavior" for a discussion of dengue.) The incidence of Japanese encephalitis, another mosquito-borne disease that accounts for almost 30,000 human cases and approximately 7,000 deaths annually in Asia, is closely associated with flooding of fields for rice growing. Outbreaks of Rift Valley fever in some parts of Africa have been associated with dam building as well as with periods of heavy rainfall (19). In the outbreaks of Rift Valley fever in Mauritania in 1987, the human cases occurred in villages near dams on the Senegal River. The same effect has been documented with other infections that have aquatic hosts, such as schistosomiasis.

Because humans are important agents of ecological and environmental change, many of these factors are anthropogenic. Of course, this is not always the case, and natural environmental changes, such as climate or weather anomalies, can have the same effect. The outbreak of hantavirus pulmonary syndrome in the southwestern United States in 1993 is an example. It is likely that the virus has long been present in mouse populations but an unusually mild and wet winter and spring in that area led to an increased rodent population in the spring and summer and thus to greater opportunities for people to come in contact with infected rodents (and, hence, with the virus); it has been suggested that the weather anomaly was due to large-scale climatic effects (20). The same causes may have been responsible for outbreaks of hantaviral disease in Europe at approximately the same time (21,22). With cholera, it has been suggested that certain organisms in marine environments are natural reservoirs for cholera vibrios, and that large scale effects on ocean currents may cause local increases in the reservoir organism with consequent flare-ups of cholera (23).

### Changes in Human Demographics and Behavior

Human population movements or upheavals, caused by migration or war, are often important factors in disease emergence. In many parts of the world, economic conditions are encouraging the mass movement of workers from rural areas to cities. The United Nations has estimated that, largely as a result of continuing migration, by the year 2025, 65% of the world population (also expected to be larger in absolute numbers), including 61% of the population in developing regions, will live in cities (24). As discussed above for HIV, rural urbanization allows infections arising in isolated rural areas, which may once have remained obscure and localized, to reach larger populations. Once in a city, the newly introduced infection would have the opportunity to spread locally among the population and could also spread further along highways and interurban transport routes and by airplane. HIV has been, and in Asia is becoming, the best known beneficiary of this dynamic, but many other diseases, such as dengue, stand to benefit. The frequency of the most severe form, dengue hemorrhagic fever, which is thought to occur when a person is sequentially infected by two types of dengue virus, is increasing as different dengue viruses have extended their range and now overlap (25). Dengue hemorrhagic fever is now common in some cities in Asia, where the high prevalence of infection is attributed to the proliferation of open containers needed for water storage (which also provide breeding grounds for the mosquito vector) as the population size exceeds the infrastructure (19). In urban environments, rain-filled tires or plastic bottles are often breeding grounds of choice for mosquito vectors. The resulting mosquito population boom is complemented by the high human population density in such situations, increasing the chances of stable transmission cycles between infected and susceptible persons. Even in industrialized countries, e.g., the United States, infections such as tuberculosis can spread through high-population density settings (e.g., day care centers or prisons) (12,26-28).

Human behavior can have important effects on disease dissemination. The best known examples are sexually transmitted diseases, and the ways in which such human behavior as sex or intravenous drug use have contributed to the emergence of HIV are now well known. Other factors responsible for disease emergence are influenced by a variety of human actions, so human behavior in the broader sense is also very important. Motivating appropriate individual behavior and constructive action, both locally and in a larger scale, will be essential for controlling emerging infections. Ironically, as AIDS prevention efforts have demonstrated, human behavior remains one of the weakest links in our scientific knowledge.

### International Travel and Commerce

The dissemination of HIV through travel has already been mentioned. In the past, an infection introduced into people in a geographically isolated area might, on occasion, be brought to a new place through travel, commerce, or war (8). Trade between Asia and Europe, perhaps beginning with the silk route and continuing with the Crusades, brought the rat and one of its infections, the bubonic plague, to Europe. Beginning in the 16th and 17th centuries, ships bringing slaves from West Africa to the New World also brought yellow fever and its mosquito vector, *Aedes aegypti*, to the new territories. Similarly, smallpox escaped its Old World origins to wreak new havoc in the New World. In the 19th century, cholera had similar opportunities to spread from its probable origin in the Ganges plain to the Middle East and, from there, to Europe and much of the remaining world. Each of these infections had once been localized and took advantage of opportunities to be carried to previously unfamiliar parts of the world.

Similar histories are being repeated today, but opportunities in recent years have become far richer and more numerous, reflecting the increasing volume, scope, and speed of traffic in an increasingly mobile world. Rats have carried hantaviruses virtually worldwide (29). *Aedes albopictus* (the Asian tiger mosquito) was introduced into the United States, Brazil, and parts of Africa in shipments of used tires from Asia (30). Since its introduction in 1982, this mosquito has established itself in at least 18 states of the United States and has acquired local viruses including Eastern equine encephalomyelitis (31), a cause of serious disease. Another mosquito-borne disease, malaria, is one of the most frequently imported diseases in non-endemic-disease areas, and cases of “airport malaria” are occasionally identified.

A classic bacterial disease, cholera, recently entered both South America (for the first time this century) and Africa. Molecular typing shows the South American isolates to be of the current pandemic strain (32), supporting the suggestion that the organism was introduced in contaminated bilge water from an Asian freighter (33). Other evidence indicates that cholera was only one of many organisms to travel in ballast water; dozens, perhaps hundreds, of species have been exchanged between distant places through this means of transport alone. New bacterial strains, such as the recently identified *Vibrio cholerae* O139, or an epidemic strain of *Neisseria meningitidis* (34,35) (also examples of microbial adaptation and change) have disseminated rapidly along routes of trade and travel, as have antibiotic-resistant bacteria (5,36).

### Technology and Industry

High-volume rapid movement characterizes not only travel, but also other industries in modern society. In operations, including food production, that process or use products of biological origin, modern production methods yield increased efficiency and reduced costs but can increase the chances of accidental contamination and amplify the effects of such contamination. The problem is further compounded by globalization, allowing the opportunity to introduce agents from far away. A pathogen present in some of the raw material may find its way into a large batch of final product, as happened with the contamination of hamburger meat by *E. coli* strains causing hemolytic uremic syndrome (37). In the United States the implicated *E. coli* strains are serotype O157:H7; additional serotypes have been identified in other countries. Bovine spongiform encephalopathy (BSE), which emerged in Britain within the last few years, was likely an interspecies transfer of scrapie from sheep to cattle (38) that occurred when changes in rendering processes led to incomplete inactivation of scrapie agent in sheep byproducts fed to cattle (39).

The concentrating effects that occur with blood and tissue products have inadvertently disseminated infections unrecognized at the time, such as HIV and hepatitis B and C. Medical settings are also at the front line of exposure to new diseases, and a number of infections, including many emerging infections, have spread nosocomially in health care settings (Table 2). Among the numerous examples, in the outbreaks of Ebola fever in Africa many of the secondary cases were hospital acquired, most transmitted to other patients through contaminated hypodermic apparatus, and some to the health care staff by contact. Transmission of Lassa fever to health care workers has also been documented.

On the positive side, advances in diagnostic technology can also lead to new recognition of agents that are already widespread. When such agents are newly recognized, they may at first often be labeled, in some cases incorrectly, as emerging infections. Human herpesvirus 6 (HHV-6) was identified only a few years ago, but the virus appears to be extremely widespread (40) and has recently been implicated as the cause of roseola (exanthem subitum), a very common childhood disease (41). Because roseola has been known since at least 1910, HHV-6 is likely to have been common for decades and probably much longer. Another recent example is the bacterium *Helicobacter pylori*, a probable cause of gastric ulcers (42) and some cancers (43,44). We have lived with these diseases for a long time without knowing their cause. Recognition of the agent is often advantageous, offering new promise of controlling a previously intractable disease, such as treating gastric ulcers with specific antimicrobial therapy.

### Microbial Adaptation and Change

Microbes, like all other living things, are constantly evolving. The emergence of antibiotic-resistant bacteria as a result of the ubiquity of antimicrobials in the environment is an evolutionary lesson on microbial adaptation, as well as a demonstration of the power of natural selection. Selection for antibiotic-resistant bacteria (5,36) and drug-resistant parasites has become frequent, driven by the wide and sometimes inappropriate use of antimicrobial drugs in a variety of applications (27,45,46). Pathogens can also acquire new antibiotic resistance genes from other, often nonpathogenic, species in the environment (36), selected or perhaps even driven by the selection pressure of antibiotics.

Many viruses show a high mutation rate and can rapidly evolve to yield new variants (47). A classic example is influenza (48). Regular annual epidemics are caused by “antigenic drift” in a previously circulating influenza strain. Achange in an antigenic site of a surface protein, usually the hemagglutinin (H) protein, allows the new variant to reinfect previously infected persons because the altered antigen is not immediately recognized by the immune system.

On rare occasions, perhaps more often with nonviral pathogens than with viruses (49), the evolution of a new variant may result in a new expression of disease. The epidemic of Brazilian purpuric fever in 1990, associated with a newly emerged clonal variant of *Hemophilus influenzae*, biogroup *aegyptius*, may fall into this category. It is possible, but not yet clear, that some recently described manifestations of disease by group A *Streptococcus*, such as rapidly invasive infection or necrotizing fasciitis, may also fall into this category.

### Breakdown of Public Health Measures and Deficiencies in Public Health Infrastructure

Classical public health and sanitation measures have long served to minimize dissemination and human exposure to many pathogens spread by traditional routes such as water or preventable by immunization or vector control. The pathogens themselves often still remain, albeit in reduced numbers, in reservoir hosts or in the environment, or in small pockets of infection and, therefore, are often able to take advantage of the opportunity to reemerge if there are breakdowns in preventive measures.

Reemerging diseases are those, like cholera, that were once decreasing but are now rapidly increasing again. These are often conventionally understood and well recognized public health threats for which (in most cases) previously active public health measures had been allowed to lapse, a situation that unfortunately now applies all too often in both developing countries and the inner cities of the industrialized world. The appearance of reemerging diseases may, therefore, often be a sign of the breakdown of public health measures and should be a warning against complacency in the war against infectious diseases.

Cholera, for example, has recently been raging in South America (for the first time in this century) (50) and Africa. The rapid spread of cholera in South America may have been abetted by recent reductions in chlorine levels used to treat water supplies (34). The success of cholera and other enteric diseases is often due to the lack of a reliable water supply. These problems are more severe in developing countries, but are not confined to these areas. The U.S. outbreak of waterborne *Cryptosporidium* infection in Milwaukee, Wisconsin, in the spring of 1993, with over 400,000 estimated cases, was in part due to a nonfunctioning water filtration plant (51); similar deficiencies in water purification have been found in other cities in the United States (52).

### For our Future

In his accompanying article, Dr. David Satcher discusses the history of infectious diseases and the many infections that, from the dawn of history to the present, have traveled with the caravans and followed the invading armies. The history of infectious diseases has been a history of microbes on the march, often in our wake, and of microbes that have taken advantage of the rich opportunities offered them to thrive, prosper, and spread. And yet the historical processes that have given rise to the emergence of “new” infections throughout history continue today with unabated force; in fact, they are accelerating, because the conditions of modern life ensure that the factors responsible for disease emergence are more prevalent than ever before. Speed of travel and global reach are further borne out by studies modeling the spread of influenza epidemics (53) and HIV (54,55).

Humans are not powerless, however, against this relentless march of microbes. Knowledge of the factors underlying disease emergence can help focus resources on the key situations and areas worldwide (3,4) and develop more effective prevention strategies. If we are to protect ourselves against emerging diseases, the essential first step is effective global disease surveillance to give early warning of emerging infections (3,12,13,56). This must be tied to incentives, such as national development, and eventually be backed by a system for an appropriate rapid response. World surveillance capabilities are critically deficient (12,56,57). Efforts, such as the CDC plan (13), now under way in the United States and internationally to remedy this situation are the essential first steps and deserve strong support. Research, both basic and applied, will also be vital.

### This Journal and the "Perspectives" Section

Early warning of emerging and reemerging infections depends on the ability to identify the unusual as early as possible. Information is, therefore, essential. Hence this journal, which is intended as a peer-reviewed forum for the discussion of concepts and examples relevant to emerging infectious diseases and their causes, and to provide a channel for field reports and observations on emerging infections. The "Perspectives" section will provide general overviews dealing with factors in disease emergence, conceptual syntheses of information, approaches for studying or predicting emerging infections, and analyses that shed light on how and why infections emerge, and how they may be anticipated and prevented. Submissions for this section are warmly invited. In coming issues, Perspectives will deal in greater detail with many of the factors discussed in this overview article, and with ways to dissect steps in the emergence process. Discussion of technologies that are broadly applicable to the identification or control of emerging diseases are also appropriate for this section. Case studies are welcome if they are used to develop broader lessons.

### Acknowledgments

I thank Dr. John La Montagne, NIAID, for helpful discussions. Supported by NIH grant RR 03121 (from CMP), US DHHS.

*Dr. Morse, "Perspectives" section editor of this journal, is assistant professor of virology at The Rockefeller University, New York, N.Y. He chaired the NIH Conference on Emerging Viruses (May 1989) and was a member of the committee on Emerging Microbial Threats to Health (and chaired its Task Force on Viruses), convened by the Institute of Medicine, National Academy of Sciences (ref. 12).*

### References

1. Morse SS, Schluederberg A. Emerging viruses: the evolution of viruses and viral diseases. J Infect Dis 1990;162:1-7.
2. Morse SS. Examining the origins of emerging viruses. In: Morse SS, ed. Emerging viruses. New York: Oxford University Press, 1993:10-28.
3. Morse SS. Regulating viral traffic. Issues Sci Technol 1990;7:81-4.
4. Morse SS. Emerging viruses: defining the rules for viral traffic. Perspect Biol Med 1991;34:387-409.
5. Soares S, Kristinsson KG, Musser JM, Tomasz A. Evidence for the introduction of a multiresistant clone of serotype 6B *Streptococcus pneumoniae* from Spain to Iceland in the late 1980s. J Infect Dis 1993;168:158-63.
6. Rogers DJ, Packer MJ. Vector-borne diseases, models, and global change. Lancet 1993;342:1282-4.
7. Fiennes RW. Zoonoses and the origins and ecology of human disease. London: Academic Press, 1978.
8. McNeill WH. Plagues and peoples. New York: Anchor Press/ Doubleday, 1976.
9. Myers G, MacInnes K, Korber B. The emergence of simian/human immunodeficiency viruses. AIDS Res Hum Retroviruses 1992;8:373-86.
10. Allan JS, Short M, Taylor ME, et al. Species-specific diversity among simian immunodeficiency viruses from African green monkeys. J Virol 1991;65:2816-28.
11. Gao F, Yue L, White AT, et al. Human infection by genetically diverse SIVSM-related HIV-2 in West Africa. Nature 1992;358:495-9.
12. Institute of Medicine. Emerging infections: Microbial threats to health in the United States (Lederberg J, Shope RE, Oaks SC Jr, eds). Washington, DC: National Academy Press, 1992.
13. Centers for Disease Control and Prevention. Addressing emerging infectious disease threats: a prevention strategy for the United States. Atlanta, Georgia: US Dept of Health and Human Services, Public Health Service, 1994.
14. Barbour AG, Fish D. The biological and social phenomenon of Lyme disease. Science 1993;260:1610-6.
15. Johnson KM. Emerging viruses in context: an overview of viral hemorrhagic fevers. In: Morse SS, ed. Emerging viruses. New York: Oxford University Press, 1993:46-7.
16. Webster RG, Bean WJ, Gorman OT, Chambers TM, Kawaoka Y. Evolution and ecology of influenza A viruses. Microbiol Rev 1992;56:152-79.
17. Scholtissek C, Naylor E. Fish farming and influenza pandemics. Nature 1988;331:215.
18. World Health Organization. Geographical distribution of arthropod-borne diseases and their principal vectors. Geneva: World Health Organization (WHO/VBC/89.967), 1989:138-48.
19. Monath TP. Arthropod-borne viruses. In: Morse SS, ed. Emerging viruses. New York: Oxford University Press, 1993.
20. Levins R, Epstein PR, Wilson ME, Morse SS, Slooff R, Eckardt I. Hantavirus disease emerging. Lancet 1993;342:1292.
21. Le Guenno B, Camprasse MA, Guilbaut JC, Lanoux P, Hoen B. Hantavirus epidemic in Europe, 1993. Lancet 1994;343:114-5.
22. Rollin PE, Coudrier D, Sureau P. Hantavirus epidemic in Europe, 1993. Lancet 1994;343:115-6.

23. Epstein, PR, Ford TE, Colwell RR. Marine ecosystems. Lancet 1993;342:1216-9.
24. United Nations. World urbanization prospects, 1990. New York: United Nations, 1991.
25. Gubler DJ, Trent DW. Emergence of epidemic dengue/dengue hemorrhagic fever as a public health problem in the Americas. Infectious Agents and Disease 1993;26:383-93.
26. Krause RM. The origin of plagues: old and new. Science 1992;257:1073-8.
27. Bloom BR, Murray CJL. Tuberculosis: commentary on a reemergent killer. Science 1992;257:1055-64.
28. Hoge CW, Reichler MR, Dominguez EA, *et al*. An epidemic of pneumococcal disease in an overcrowded, inadequately ventilated jail. N Engl J Med 1994;331:643-8.
29. LeDuc JW, Childs JE, Glass GE. The hantaviruses, etiologic agents of hemorrhagic fever with renal syndrome: a possible cause of hypertension and chronic renal disease in the United States. Annu Rev Public Health 1992;13:79-98.
30. Centers for Disease Control and Prevention. *Aedes albopictus* introduction into continental Africa, 1991. MMWR 1991;40:836-8.
31. Centers for Disease Control and Prevention. Eastern equine encephalitis virus associated with *Aedes albopictus*—Florida, 1991. MMWR 1992;41:115, 121.
32. Wachsmuth IK, Evins GM, Fields PI, *et al*. The molecular epidemiology of cholera in Latin America. J Infect Dis 1993;167:621-6.
33. Anderson C. Cholera epidemic traced to risk miscalculation [News]. Nature 1991;354:255.
34. Moore PS. Meningococcal meningitis in sub-Saharan Africa: a model for the epidemic process. Clin Infect Dis 1992;14:515-25.
35. Moore PS, Broome CV. Cerebrospinal meningitis epidemics. Sci Am 1994;271(5):38-45.
36. Davies J. Inactivation of antibiotics and the dissemination of resistance genes. Science 1994;264:375-82.
37. Centers for Disease Control and Prevention. Update: multistate outbreak of *Escherichia coli* O157:H7 infections from hamburgers—western United States, 1992-1993. MMWR 1993;42:258-63.
38. Morse SS. Looking for a link. Nature 1990;344:297.
39. Wilesmith JW, Ryan JBM, Atkinson MJ. Bovine spongiform encephalopathy: epidemiological studies on the origin. Vet Rec 1991;128:199-203.
40. Inoue N, Dambaugh TR, Pellett PE. Molecular biology of human herpesviruses 6A and 6B. Infectious Agents and Disease 1993;26:343-60.
41. Yamanishi K, Okuno T, Shiraki K, *et al*. Identification of human herpesvirus-6 as a causal agent for exanthem subitum. Lancet 1988;i:1065-7.
42. Peterson WL. *Helicobacter pylori* and peptic ulcer disease. N Engl J Med 1991;324:1043-8.
43. Nomura A, Stemmermann GN, Chyou P-H, Kato I, Perez-Perez GI, Blaser MJ. *Helicobacter pylori* infection and gastric carcinoma among Japanese Americans in Hawaii. N Engl J Med 1991;325:1132-6.
44. Parsonnet J, Friedman GD, Vandersteen DP, *et al*. *Helicobacter pylori* infection and the risk of gastric carcinoma. N Engl J Med 1991;325:1127-31.
45. Cohen ML. Epidemiology of drug resistance: implications for a post-antimicrobial era. Science 1992;257:1050-5.
46. Neu HC. The crisis in antibiotic resistance. Science 1992;257:1064-72.
47. Domingo E, Holland JJ. Mutation rates and rapid evolution of RNA viruses. In: Morse SS, ed. The evolutionary biology of viruses. New York: Raven Press, 1994:161-84.
48. Kilbourne ED. The molecular epidemiology of influenza. J Infect Dis 1978;127:478-87.
49. Morse SS. Toward an evolutionary biology of viruses. In: Morse SS, ed. The evolutionary biology of viruses. New York: Raven Press, 1994:1-28.
50. Glass RI, Libel M, Brandling-Bennett AD. Epidemic cholera in the Americas. Science 1992;265:1524-5.
51. MacKenzie WR, Hoxie NJ, Proctor ME, *et al*. A massive outbreak in Milwaukee of *Cryptosporidium* infection transmitted through the water supply. N Engl J Med 1994;331:161-7.
52. Centers for Disease Control and Prevention. Assessment of inadequately filtered public drinking water—Washington, D.C., December 1993. MMWR 1994;43:661-3.
53. Longini IM Jr, Fine PEM, Thacker SB. Predicting the global spread of new infectious agents. Am J Epidemiol 1986;123:383-91.
54. Flahault A, Valleron AJ. HIV and travel, no rationale for restrictions. Lancet 1990;336:1197-8.
55. Flahault A, Valleron AJ. A method for assessing the global spread of HIV-1 infection based on air travel. Mathematical Population Studies 1992;3:161-71.
56. Henderson DA. Surveillance systems and intergovernmental cooperation. In: Morse SS, ed. Emerging viruses. New York: Oxford University Press, 1993:283-9.
57. Berkelman RL, Bryan RT, Osterholm MT, LeDuc JW, Hughes JM. Infectious disease surveillance: a crumbling foundation. Science 1994;264:368-70.
